# Establishment of Metabolism and Transport Pathways in the Rodent and Human Fetal Liver

**DOI:** 10.3390/ijms141223801

**Published:** 2013-12-06

**Authors:** Jamie E. Moscovitz, Lauren M. Aleksunes

**Affiliations:** 1 Department of Pharmacology and Toxicology, Rutgers University Ernest Mario School of Pharmacy, 170 Frelinghuysen Rd., Piscataway, NJ 08854, USA; E-Mail: jamie.moscovitz@rutgers.edu; 2 Environmental and Occupational Health Sciences Institute, 170 Frelinghuysen Rd., Piscataway, NJ 08854, USA

**Keywords:** fetal, metabolism, transport, regulation, prenatal, phase I, phase II, Abc, Slc, ontogeny

## Abstract

The ultimate fate of drugs and chemicals in the body is largely regulated by hepatic uptake, metabolism, and excretion. The liver acquires the functional ability to metabolize and transport chemicals during the perinatal period of development. Research using livers from fetal and juvenile rodents and humans has begun to reveal the timing, key enzymes and transporters, and regulatory factors that are responsible for the establishment of hepatic phase I and II metabolism as well as transport. The majority of this research has been limited to relative mRNA and protein quantification. However, the recent utilization of novel technology, such as RNA-Sequencing, and the improved availability and refinement of functional activity assays, has begun to provide more definitive information regarding the extent of hepatic drug disposition in the developing fetus. The goals of this review are to provide an overview of the early regulation of the major phase I and II enzymes and transporters in rodent and human livers and to highlight potential mechanisms that control the ontogeny of chemical metabolism and excretion pathways.

## 1. Introduction

Xenobiotics that enter the body commonly undergo metabolism by phase I and/or phase II enzymes in the liver. Hepatic phase I reactions can include oxidation, reduction, or hydrolysis, and are predominantly performed by cytochrome P450 (Cyp), flavin-containing monooxygenase (Fmo), and carboxylesterase (Ces) enzymes. Phase II reactions include the addition of an endogenous compound to a xenobiotic by a transferase such as glutathione *S*-transferase (Gst), sulfotransferase (Sult), UDP-glucuronosyl transferase (Ugt), and *N*-acetyl transferase (Nat). In order for large and/or charged molecules to gain access to metabolizing enzymes, transporters may be required to bring chemicals from sinusoidal blood into hepatocytes. In addition, transporters mediate the removal of chemicals from cells, in particular, conjugated xenobiotics following phase II metabolism. Two important superfamilies of transporters include the solute carrier (Slc) and ATP-binding cassette (Abc) transporters, which are generally involved in the uptake and efflux of substances, respectively. Similar to metabolic enzymes, each transporter isoform can have unique specificity and affinity for substrates. Together, metabolizing enzymes and transporters account for a large proportion of the biotransformation and disposition of chemicals in the body.

The fields of pharmacology, toxicology, and medicine have benefited greatly from advancements in understanding the developmental profiles of hepatic phase I and II drug metabolism and transport. These data have aided in chemical risk assessment, exposure modeling, and age-appropriate pharmacotherapy. In toxicology, the knowledge of the developmental profiles for disposition pathways has helped to identify human populations that are susceptible, as well as resistant, to the effects of different environmental chemicals and drugs. Still, more research is needed to understand the ontogeny and underlying regulation of these processes.

The fetal period of development is characterized by tissue differentiation and growth, and is designated as gestation day 17 to birth (days 19–22) in mice and rats, and day 56 to birth (day 267) in humans. Between gestation days 9.5 and 15 in mice, as well as days 60 and 195 in humans, the liver is the predominant hematopoietic site in the body. Following this period, primary erythropoiesis shifts to the bone marrow, while hematopoiesis gradually decreases in the liver. Around gestation day 13, hepatoblasts mature into functional rodent hepatocytes and begin the early formation of a biliary network, which continues through birth.

It was first observed in the 1950s that the drug metabolizing capabilities of the newborn rabbit liver are not mature at birth because of a differing enzyme profile [1]. Subsequently, we have learned that drug metabolizing enzyme and transporter expression in the fetal and neonatal periods follow several developmental patterns, and vary for each subfamily and isoform. Resources to understand the functioning of the human fetal liver are limited, and thus rodent models have been primarily used to study this important stage of development. In recent years, however, the availability of human fetal tissues has increased, providing greater insight into the ontogeny of drug metabolism and transport across species.

This review article provides a broad overview of the rodent and human studies that characterize the regulation of phase I and II enzymes and transporters during fetal liver development. Lowercase letter abbreviations have been used to denote rodent gene and protein isoforms and uppercase letter abbreviations refer to human orthologs. While the majority of studies have focused on mRNA regulation, there are some data regarding functional changes during hepatic development that will be discussed. In this review, attention will be placed upon regulatory pathways, including transcriptional and epigenetic factors, that contribute to the programming of biotransformation and excretion.

## 2. Phase I Metabolism

While phase I drug metabolism in the fetal liver is relatively immature compared to the adult liver, it does exist primarily as a result of Cyp enzyme activity. Although other phase I enzymes may be present before birth, their expression is quite low. The mRNA and/or protein expression of rodent and human aldehyde dehydrogenase (Aldh) [2,3] and Fmo isoforms [3–6] in fetal livers is much less than observed in adults (Table 1). Likewise, the expression and/or activity of Ces enzymes is rather diminished [3,7,8]. While there are exceptions, such as human FMO1, which has been detected in second trimester fetal livers [5,6], the majority of phase I metabolism during early development appears to be Cyp-mediated biotransformation and thus, most research in this field has focused on Cyp enzymes.

### 2.1. Cytochrome P450 Enzymes

CYP enzymes oxidize a variety of exogenous and endogenous substrates. They are highly expressed in the liver, as well as the lungs and kidneys. The mouse liver expresses 31 of the 102 total Cyp enzymes that have been identified [9,10]. This is the largest number of Cyp isoforms present in a single mouse tissue. In contrast, 57 CYP enzymes have been identified in humans [10]. While not all mouse Cyp isoforms have been shown to metabolize specific substrates, more than half exhibit sequence homology to human isoforms [10,11]. The fetal liver mRNA, protein and/or activity of Cyp/CYP enzymes in rodents and humans has been characterized relative to adult levels and summarized in Table 2.

#### 2.1.1. Mouse Cyp Regulation

In general, gene expression of most Cyps is low in the mouse fetal liver, and gradually increases with advancing age. mRNA and protein for many Cyp isoforms can be subsequently detected at postnatal days 15 to 20 and beyond, when their expression is equivalent to that of an adult mouse liver [2,11]. Notably, Cyp3a16 and 3a41b transcripts are enriched perinatally (two days before birth through 5 days after birth), while adult mRNA levels of these isoforms are detectable but low [11,14]. While these two isoforms are preferentially higher during this developmental window, the absolute abundance of their mRNAs is lower than other Cyp isoforms found in the fetal liver (such as Cyp2d26).

#### 2.1.2. Rat Cyp Regulation

Several isoforms that exhibit elevated mRNA expression and/or activity in the rat fetal period, such as Cyp1a1, 3a2 and 4a1, have been identified [7]. Despite the fact that mRNA levels of Cyp1a1 have been shown to be 1.5-fold higher in the fetal liver than the adult liver, no activity for Cyp1a1 in fetal tissues has been measurable (assessed by oxidative deethylation of 7-ethoxyresorufin) [7]. Interestingly, up-regulation of fetal hepatic mRNA for Cyp1a1, as well as Cyp1a2, 2b1, and 3a2 is seen with exposure of pregnant rats to the environmental contaminant, polybrominated diphenyl ether, BDE-99 [25]. In this study, elevated Cyp mRNA following BDE-99 treatment coincided with generation of lipid peroxides and increased activity of catalase. These data suggest that while Cyps are modestly expressed in the rat fetal liver, they may be inducible by xenobiotics, which could impact hormone signaling and endocrine programming during development.

Sex-specific differences in the fetal establishment of Cyp3a2 and 4a1 mRNA expression and activity have been demonstrated. Cyp3a2 is a male-specific Cyp isoform that metabolizes a variety of drugs and steroids, while Cyp4a1 participates in hepatic lipid metabolism. Compared to levels found in adult rat livers, Cyp3a2 mRNA is higher in female fetuses, and lower in male fetuses. It should be noted that the activity for Cyp3a2 is negligible in both sexes, as quantified by *N*-ethylmorphine *N*-demethylation. Fetal Cyp4a1 mRNA transcripts are equivalent to that of the adult liver, whereas activity measured by lauric acid ω-hydroxylation is increased by 190% in female rat fetuses and decreased by 70% in male rat fetuses relative to the adult activity of the respective sex [7]. Additional studies are needed to better understand the gender divergent regulation of fetal Cyp enzymes and the importance of these differences for lipid and steroid metabolism.

#### 2.1.3. Human CYP Regulation

Total CYP enzyme protein in the human fetal liver has been reported as 30% to 60% of adults [26–28]. Of these CYPs, the human CYP3A subfamily is critical for xenobiotic metabolism, and is likely the best characterized class of Phase I enzymes. CYP3A7 accounts for 30% of total fetal CYP content, and exhibits its highest expression on postnatal days 1 to 7 [19]. Fetal CYP3A7 mRNA and protein expression, as well as catalytic activity, have been demonstrated by several studies and discussed in a prior review [20,21,29]. Following this early postnatal period, there is a change from CYP3A7-predominant to CYP3A4-predominant activity in the liver. This is illustrated by a higher propensity for 16-alpha hydroxylation of dehydroepiandrosterone by CYP3A7 during gestation and a transition to testosterone 6-beta hydroxylase activity associated with CYP3A4 after birth [20]. Prior to this shift away from CYP3A7-mediated metabolism, the fetus has 10% of the adult content of CYP3A4 [19]. The related CYP isoform, CYP3A5, exhibits high inter-individual differences in protein, with no change between the fetal, perinatal, and postnatal periods [19,21]. Other prominent human CYP isoforms, such as CYP2D6 and CYP2E1, have low fetal protein (~10% of adult values) [29–31], and do not typically increase until adolescence. Both CYP2D6 and CYP2E1 also exhibit high inter-individual differences, similar to CYP3A5. While CYP2C19 protein and activity is consistently low throughout the fetal period (10%–15% adult levels), CYP2C9 protein and activity is 1% of adult levels in the first trimester, increasing to 30% during the second and third trimesters [18,29]. CYP1A2 is the final major drug metabolizing isoform to develop in the human liver [12]. CYP1A2 protein is undetectable in the fetal liver and increases modestly to 50% of adult levels around one year of age [12].

Although many orthologs exhibit similar fetal enzymatic profiles in both rodent and human livers, there are some exceptions. Whereas CYP3A7 “switches” with CYP3A4 by 1 to 2 years of age in humans, the mouse ortholog Cyp3a16 is relatively stable and has sustained expression through adolescence when there is a developmental shift to Cyp3a11, the adult isoform [19–21,32,33]. Additional research is needed to better understand the mechanism(s) underlying the differences in CYP3A/Cyp3a ontogeny between species.

#### 2.1.4. Bile Acid-Related Cyps

Bile acid enzyme mRNAs of the classic synthetic pathway, Cyp7a1 and Cyp8b1, are low two days before birth in mice, and increase by approximately 14- and 8-fold at parturition, respectively [22]. This up-regulation at birth correlates with a surge in the major bile acid of the classic pathway, cholic acid, as well as its conjugates. It is hypothesized that the up-regulation of bile acid synthesis enzymes is due, in part, to activation of the liver X receptor (Lxr), as well as suppression of fibroblast growth factor 15 (Fgf15), which together promote transcription of Cyp7a1 [22]. Conversely, enzymes of the alternative synthesis pathway, namely Cyp27a1 and Cyp7b1, are less than 3% of adult levels two days before birth, and stay low until days 20 to 30, and are likely under different regulatory control mechanisms [22].

### 2.2. Carboxylesterase Enzymes

CES enzymes hydrolyze chemicals with ester, thioester, or amide bonds. They can be localized to the endoplasmic reticulum as well as the cytosol. CES enzymes are highly expressed in the liver, small intestine, kidneys, and lungs. Mouse Ces1 mRNA comprises 58% of total adult liver Ces transcripts, while Ces2 and Ces3 families are also present [3]. Isoforms of all three families are low in the fetal liver as compared to the adult, with gradual increases in enrichment from birth to day 45 [3]. In addition, rat fetal hepatic microsomes exhibit ~15% of the hydrolysis of the model Ces substrate *p*-nitrophenyl acetate, as compared to microsomes generated from adult animals [7]. Considerable work has documented the increasing expression and activity of CESs with age, across multiple species including mouse, rat, and human. These studies have focused on the role of CES in the detoxification of different insecticides and pesticides, including pyrethroids such as deltamethrin and organophosphates such as parathion [8,34–37]. Because of the delayed establishment of CES-mediated metabolism, juvenile mammals can have increased systemic exposure to environmental chemicals and drugs that may lead to significant toxicity.

### 2.3. Absolute Expression of Phase I Enzymes in Fetal and Neonatal Mouse Liver

In recent years, researchers have begun to apply novel technologies to understand the developmental regulation of drug metabolism pathways in mice. This includes the use of RNA-Sequencing, which is a next generation sequencing technology that simultaneously quantifies RNA transcripts and variants. Using RNA-Sequencing, two recent studies have comprehensively quantified the expression of phase I enzymes in the fetal mouse liver (two days prior to birth) [3,14]. The most abundant transcripts include Cyps (35%), followed by Aldhs (25%), Aldo-keto reductases (Akrs, 21%), and Alcohol dehydrogenases (Adhs, 10%) [3]. Cyp2d26 accounts for 40% of the total Cyp mRNAs two days prior to birth, followed by Cyp2c68 (8%), and Cyp2d10 (7%), with all other isoforms at less than 6% [14]. In addition to these Cyp enzymes, a number of other phase I enzymes are enriched including Akr1a4, Adh5, Aldh2, and Aldh4a1 [3,14].

Recently, analysis of 12 human fetal livers has identified low (ADH7, ALDH3B2, ALD1A3, CES5A, CES3, CYP4F8, CYP26C1, CYP11B2, FMO2) and high (ADH1A, ADH6, ADH5, ALDH1A1, ALDH2, ALDH4A1, CYP3A7, CYP27A1, CYP19A1, FMO5) expression of phase I metabolizing enzymes using RNA-Sequencing [38].

### 2.4. Potential Mechanisms for the Regulation of Phase I Metabolism during Ontogeny

Several hypotheses have been presented to explain the expression patterns for drug metabolizing enzymes during different developmental windows. It has been suggested that the greatest contributor to expression patterns during these periods is the changing environment. This hypothesis is supported by changes in both rodent and human phase I enzymatic profiles regardless of the exact gestational day at birth [19,20,32]. In addition, some phase I enzymes may serve developmental functions, as opposed to xenobiotic metabolic activity, which could account for their early, albeit low expression [9,11,13,29,32]. For example, critical developmental function has been documented for Cyp1b1/CYP1B1 which is associated with proper eye formation and Cyps that participate in bile acid synthesis [9,22,39]. Similarly, there is a higher extrahepatic mRNA expression of mouse fetal Aldh enzymes critical for the synthesis of retinoic acid, though still at lower levels than adults [2]. While a number of phase I enzymes have been shown to support organogenesis and organ function in other tissues during development, there is the potential for additional isoforms to contribute to the establishment of key hepatic functions.

Gene clusters and transcription factors are also proposed as mediators that control the fetal and neonatal expression of CYPs. In some instances, genes encoding related CYP isoforms cluster on the same region of a chromosome and as a consequence share similar ontogenic expression patterns [32]. Studies have also shown that limited Cyp gene expression during fetal development coincides with low levels of upstream transcription factors, namely the nuclear receptors constitutive androstane receptor (Car), farnesoid X receptor (Fxr), pregnane X receptor (Pxr), and peroxisome proliferator-activated receptor alpha (Pparα) [22,32,40]. Supporting the hypothesis that reduced nuclear receptors lead to minimal Cyp expression in the fetus, there are parallel increases in both gene families after birth. For example, it was shown that binding of mouse hepatic nuclear factor (Hnf) 4 alpha to the promoter region of Pxr can activate nuclear receptor signaling in primary fetal hepatocytes [41]. Likewise, the absence of Hnf4α in these cells can cause the down-regulation of both Pxr and target gene Cyp3a11 [41]. While this is an interesting example, additional studies are necessary to clarify transcription factor-related mechanisms of fetal hepatic phase I enzyme down-regulation.

More recent attention has also been placed on the epigenetic regulation of drug metabolizing enzymes (reviewed in [42]). Postnatal changes in histone modifications have been associated with the transition in expression from the fetal Cyp3a16 isoform to the adult Cyp3a11 isoform during development [33]. Specifically, histone methylation patterns in the promoter region of hepatic Cyp3a16 in adult mice are indicative of gene silencing. Likewise, in mice, the Cyp1a2 promoter is hypermethylated at birth when hepatic gene expression is low. Following birth, the CpG islands in the promoter become demethylated and mRNA levels of Cyp1a2 increase [43]. These studies suggest a role for both DNA methylation and histone modifications in the ontogenic regulation of Cyp enzymes.

## 3. Phase II Metabolism

Similar to phase I enzymes, most phase II enzymes are not significantly expressed until the neonatal period, with some important exceptions (Table 3). Of the phase II genes that are expressed in the liver during this time frame, hierarchical clustering suggests that their biological functions are most related to peptide metabolism, glutathione synthesis and turnover, generation of *S*-adenosylmethionine and bile acid homeostasis [44]. Many early studies investigated the presence or absence of entire phase II enzyme families in the fetus, however this review will focus on specific phase II enzyme subfamilies and isoforms that have been identified in the fetal liver.

### 3.1. Sulfotransferase Enzymes

SULTs detoxify chemicals through conjugation with a sulfate group from 3′-phosphoadenosine-5′-phosphosulfate. Found largely in the cytosol, SULTs metabolize a wide variety of xeno- and endobiotics, including compounds containing alcohols, phenols, catechols, and amines. At this time, SULTs are thought to be the prevailing drug metabolizing enzymes in the fetal liver (reviewed in [66]). SULTs are responsible for the metabolism of hormones and steroids. In some cases, rodents and humans express distinctive isoforms based on their differing hormonal and steroidal profiles. An example of this is SULT2A1, the only SULT that conjugates hydroxysteroids in humans, while multiple isoforms accomplish this function in rodents [66].

#### 3.1.1. Mouse Sult Regulation

Sult mRNAs are expressed to varying degrees in the livers of adult mice. For example, there is high (Sult2a1/2, Sult3a1), moderate (Sult1a1, 1d1) and low enrichment (Sult1c1, Sult4a1, Sult5a1) of Sults [45]. Compared to adult mice, the livers of fetal mice only contain a subset of Sult isoforms, primarily, Sult1a1, which has a fairly broad substrate profile. Sult1a1 mRNA is present in the fetal liver and its expression continues to increase postnatally [45]. Sult1c1 mRNA is also expressed in liver two days prior to birth and decreases thereafter.

#### 3.1.2. Human SULT Regulation

Compared to mice, humans exhibit more extensive expression of SULT isoforms in the fetal *versus* the adult liver. One such isoform is SULT1A3, which sulfonates catecholamines. SULT1A3 protein is high in the fetal liver but absent from the adult liver [46]. Specifically, it has been demonstrated that hepatic SULT1A3 can conjugate the neurotransmitter dopamine during the second trimester [47]. Another example of species differences in phase II ontogeny is SULT1C1/Sult1c1. As mentioned above, Sult1c1 mRNA is present to a limited degree in the fetal mouse liver. SULT1C1 mRNA has not been significantly detected in the fetal human liver [46,67]. Rather, SULT1C2 appears to be the fetal-enriched SULT1C isoform in humans [47].

Liver has the highest level of steroid-sulfonating enzymes of any adult human tissue, however, this may not be the case in the fetus. During pregnancy, the fetal-placental unit converts dehydroepiandrosterone to a pregnancy-specific estrogen, estriol, which then circulates in maternal blood. The fetal hepatic expression of the dehydroepiandrosterone sulfotransferase, SULT2A1, is second to that of the fetal adrenal glands, although expression and catalytic activity toward its probe substrate are still significant [47,48,52,53,68]. Like SULT2A1, SULT1A1 protein is also substantial in the fetal liver [47,48]. SULT1A1 is found in hematopoietic cells and both fetal and adult hepatocytes, with higher detection in prenatal samples [46,47]. Moreover, the biological activity of both endo- and exogenous estrogens can be terminated with metabolism by SULT1E1. Fetal protein expression and activity of the estrogen SULT has also been detected at equivalent or even higher levels than in adult liver, which may reflect the need for greater hormone metabolism *in utero* or to compensate for the lack of other sulfonation enzymes prenatally [47,48,51].

### 3.2. Glutathione *S*-Transferase Enzymes

GSTs are found in both the cytosol and endoplasmic reticulum, and catalyze the addition of the tripeptide glutathione (glycine-cysteine-glutamate) to substrates. Glutathione is an important defense in the cell for neutralizing electrophiles, protecting the cell from harmful reactive oxygen species, such as free radicals and organic hydroperoxides, and in the body for drug detoxification. While the developmental pattern of glutathione conjugation by GSTs in humans varies according to the isoform, it is generally one of the last phase II enzyme classes to mature, which does not fully occur until adolescence (9 to 12 years).

#### 3.2.1. Mouse Gst Regulation

Of the 19 identified mouse Gst isoforms, the adult liver expresses cytosolic Gsta3, Gstm1, m4, and m6, Gstp1/2 (greater in males), Gstt1 and Gstz1 as well as mitochondrial Gtsk1 and microsomal MGst1 [69,70]. The fetal liver has detectable quantities of each of these isoforms, albeit with very low content (<20%) [54]. In addition, fetal mouse hepatocytes highly express Gstm5 and Mgst2 transcripts, which become barely detectable around postnatal day 15 [54].

#### 3.2.2. Rat Gst Regulation

The adult rat liver is enriched with Gsta1, a2, a8 and Gstm3, m4 protein [55]. One day prior to birth, the rat fetal liver exhibits significant Gst activity (78% of adult liver) towards the probe substrate 2-mercaptoethanol, 1-chloro-2,4-dinitrobenzene, before a transient decline postnatally and gradual increase to maximal function [55]. Using liquid chromatography, Gst subunits purified from fetal rat liver have been studied. The protein isoforms of highest proportion detected in gestational tissues were Gsta2 and Gstm3 [55]. In addition, while Gsta10 is lowly expressed in the adult liver, it is elevated in the fetal liver. Likewise, Gstp7 is present in the fetal liver and barely detectable after birth [55].

#### 3.2.3. Human GST Regulation

Little is known about the amount and activity of GST enzymes in the fetal human liver. A small study with tissues from two first trimester pregnancies (an embryo at 8 weeks gestation and a fetus at 13 weeks gestation) has characterized several GST subfamilies by western blot. Whereas GSTP1 was the most highly expressed GST protein throughout the fetus, including the liver, GSTA protein was the second most abundant isoform specifically found in the liver [56]. The same study also found significant protein levels of GSTM1 in the fetal liver [56]. A different study comprised of 61 prenatal samples from 10 weeks of gestation and older found that similar to fetal mouse liver, human GSTZ1 protein is marginally expressed and minimally active, with a significant increase seen at birth [71].

### 3.3. UDP-Glucuronosyltransferase Enzymes

UGTs are localized to the endoplasmic reticulum, and account for nearly half of total hepatic conjugation reactions in the adult [72]. UGTs catalyze the addition of glucuronic acid to a wide variety of small hydrophobic molecules. In both rodents and humans, UGTs are repressed in the fetal liver. For example, human fetal livers (first trimester) have 13 to 36 times less UGT2B mRNA than adult livers [61]. Rat Ugt1a6 is one exception to this trend. Hepatic Ugt1a6 protein is first detected 5 days prior to birth and is the most highly expressed fetal Ugt near parturition [58]. Kishi *et al*. (2008) have proposed that there is a “switch” at birth from the prenatally-enriched rat Ugt1a6 to the postnatally-enhanced rat Ugt1a1 isoform. This perinatal shift is apparent in the change of activity from phenol glucuronidation to bilirubin glucuronidation, however the molecular mechanism(s) underlying this transition is not known [58].

### 3.4. *N*-Acetyltransferase Enzymes

In the cytosol, NATs conjugate primary aryl amines with an acetyl group. Common drugs acetylated by NATs include the anti-tuberculosis drug isoniazid and sulfonamide antibiotics. Temporal development of acetylation is second to sulfation in humans, and is fully mature in the newborn infant. Adult mouse hepatocytes only express mRNA for Nat2. Similarly, although both Nat1 and Nat2 transcripts are found in the mouse fetus, fetal hepatocytes only contain Nat2 [64,73]. The ability of NAT to acetylate *p*-aminobenzoic acid has been detected in the human liver during the first trimester, however the responsible isoform(s) was not determined [65]. It is hypothesized that this phase II enzyme is critical during development because it acetylates a folate breakdown product, which ensures the turnover of folate within the embryo and prevents deficiency [73].

### 3.5. Absolute Phase II Enzyme Expression in Fetal and Neonatal Mouse Liver

Lu *et al*. (2013) recently described the ontogeny of hepatic phase II enzyme families in mice using RNA-Sequencing. Prior to birth, Gsts are the highest mRNAs detected in mouse liver. The profiles for Sults are largely unchanged between fetal and adult livers, such that Sult1a1 was shown to be the predominant isoform, accounting for more than 80% of total Sult transcripts [44]. It should be noted that there is a surge in Sult mRNAs between postnatal days 15 to 25 followed by a decline to adult levels. While there is some overlap in fetal and adult isoforms, mGst3 is the most abundant Gst mRNA in fetal liver, as opposed to mGst1 in adult liver [44]. Gstp1, t1 and m1 mRNAs are also highly expressed in fetal liver [44]. Although the absolute expression of Ugts is quite low prior to birth, some isoforms can be detected. Two days before birth, mouse livers are most highly enriched with members of the Ugt2b family, including Ugt2b34, followed by Ugt2b35, 2b36 and 2b5 [44]. At birth, Ugt1a1 expression transiently surges and accounts for more than a third of Ugt total transcripts, however Ugt2b family isoforms are extensively found in the adult livers, particularly Ugt2b5, 2b36 and 2b1 [44].

In addition to phase I drug metabolizing enzymes, RNA-Sequencing data of human fetal livers also revealed low (GSTA5, NAT2, SULT6B1, SULT1B1, SULT1C3, UGT2A1, UGT2B17, UGT1A1-10) and high (GSTA1, GSTP1, GSTO1, MGST3, MGST1, MGST2, SULT1A1, UGT2B10, UGT2B4) expression patterns of certain phase II drug metabolizing enzymes [38].

### 3.6. Potential Mechanisms for the Regulation of Phase II Metabolism during Ontogeny

Epigenetic programming has been postulated as a mechanism for regulating the ontogeny of Gsts. In a recent study, Gstz1 was studied as a target of epigenetic regulation because it is expressed most highly in the adult mouse liver, and minimally in the fetal liver. Histone 3 lysine-4 dimethylation confers an open chromatin conformation and thus accessible gene promoter regions, and is associated with active transcription. Cui *et al*. (2010) found minimal histone 3 lysine-4 dimethylation in the Gstz1 gene locus two days prior to birth, when mRNA levels are low [54]. After birth, levels of this epigenetic mark as well as Gstz1 mRNA increase. This study is an intriguing example of how epigenetic factors may repress phase II metabolism prenatally. There is great potential to expand this work to other enzyme isoforms and classes.

The expression of several of the phase II isozymes has been shown to be sex- and species-specific [45,69,74–76]. For example, Sult1a1 is significantly higher in the livers of female mice, while the opposite is true for the livers of rats [45,76]. Nevertheless, it is not known whether these differences are initiated during fetal development. Sult2a1/2 and Sult3a1 are almost exclusively present in the livers of adult female mice, while this sex difference has not been seen in fetal livers [45]. Likewise, sex differences observed for Gst mRNAs in the livers of adult mice have not been detected in mouse livers two days before birth [54]. A unique exception to this is the expression of human SULT1E1, a key mechanism for fetal estrogen inactivation. Duanmu *et al*. (2006) found early prenatal male liver expression of this isoform to be consistently higher than that of females [48]. This exception would appear to have a defining role in proper male development rather than differential drug metabolizing profiles between genders.

Other sulfotransferases involved in hormone metabolism, and also highly expressed in the fetal liver, appear to have differing patterns of enrichment throughout gestation. SULT1A1 protein is consistent throughout gestation, while SULT2A1 protein increases during the third trimester [48]. Although relatively unexplored, this observation indicates differential regulatory mechanisms of these key enzymes during development. Thus far, the nuclear receptor Pxr has been implicated in the regulation of rodent Sult2a1 [77], while PPARα has been implicated in regulation of the human isoform [78]. In addition, transcription factors Specificity Protein 1 and E-Twenty Six regulate human SULT1A1 [79]. While these transcription factors play a role in regulating Sults/SULTs in adult liver, their ability to control the ontogenic expression is unknown.

## 4. Transporters

**Figure 1.**
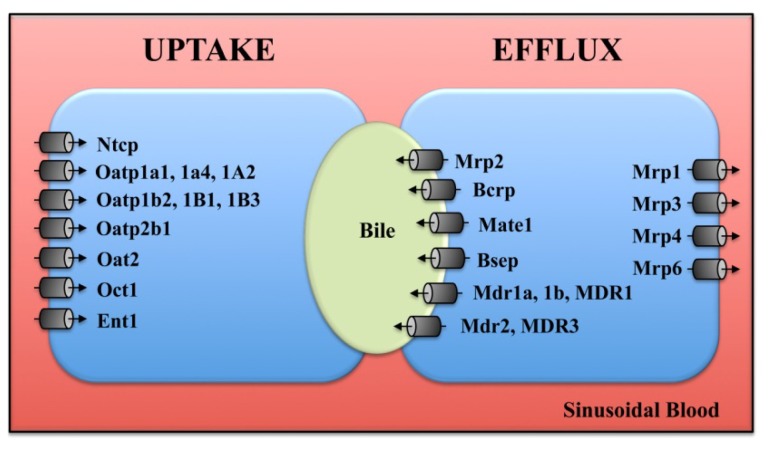
Subcellular localization of transporters in hepatocytes. The localization and orientation of uptake and efflux transporters (primarily rodent isoforms) at the sinusoidal and canalicular plasma membranes are shown.

Transport and biliary excretion are key functions of the liver that mature during development. The liver expresses a number of proteins that regulate the uptake and efflux of exogenous and endogenous organic anions and organic cations in a coordinated manner (Figure 1). Similar to phase I and II enzymes, studies investigating the fetal expression of transporters in rodents and humans have largely focused on characterization of mRNA levels during the perinatal period although studies have begun to document the ability of fetal human hepatocytes to transport bile acids and drugs (Table 4).

### 4.1. Uptake Transporters

#### 4.1.1. Mouse and Rat Uptake Transporter Regulation

The influx of positively-charged chemicals into the liver is accomplished by the organic cation transporter 1 (Oct1, Slc22a1). Oct1 mRNA is undetectable in fetal mouse hepatocytes [80,86]. It is not until birth (designated as postnatal day 0) that Oct1 mRNA is first detected at ~10% of adult transcripts [86]. A similar delay in establishment of Oct1 mRNA expression has been observed in rat liver after birth [7]. It is presumed that there is minimal organic cation transport in the prenatal liver; however, further functional studies are needed to test this hypothesis.

The liver expresses a number of organic anion transporters (Oats) and organic anion transporting polypeptides (Oatps) that are responsible for the uptake of chemicals into hepatocytes and biliary cells using sodium-independent transport. In general, Oatp mRNAs are quite low in rodent liver prior to birth [81,87,93]. mRNA of mouse Oatp2a1 (Slco2a1), which is also termed the prostaglandin transporter, is moderately expressed in late gestation that does not change during postnatal development [87]. At parturition, Oatp1b2 mRNA is induced which is followed by significant rises in Oatp1a1, 1a4, and 2b1 between postnatal days 5 and 30 in mice and rats [7,80,81,87,94]. Weak intracellular staining of Oatp1a1 protein can be observed in the newborn rat liver; however, trafficking to the sinusoidal membrane of hepatocytes is not observed until 20 days after birth [81]. In fetal rat liver, Oat3 (Slc22a8) mRNA is detected at levels higher than observed in adults, whereas Oat2 (Slc22a7) mRNA is not observed until postnatal days 5 to 15 [7,80]. Collectively, these data suggest that establishment of the majority of Oat- and Oatp-mediated transport occurs after birth and that Oat3 may be the primary organic anion uptake transporter in the fetal liver.

While the gene expression of Oatp, Oat, and Oct is relatively low in the mouse fetal liver, it should be noted that the mRNA of equilibrative nucleoside transporter 1 (Ent1, Slc29a1) has been detected [22]. Ent1 transports nucleosides that may be important in the proliferation and maturation of the liver. While Ent1 is modestly expressed in the fetal liver, this transporter continues to increase postnatally with maximal expression at postnatal day 20 [80].

### 4.2. Efflux Transporters

#### 4.2.1. Rodent and Human Efflux Transporter Regulation

Multidrug resistance (Mdr) transporters are responsible for the canalicular secretion of amphipathic molecules and cations (Mdr/Abcb1a and 1b isoforms in rodents and MDR1 in humans, also known as P-glycoprotein) and phospholipids (Mdr2/Abcb2 isoform in rodents and MDR3 in humans). Mdr1a and 1b mRNA are low in the fetal rat liver, relative to the adult [7,89]. The human MDR1 protein can be detected along the canalicular membrane of fetal livers beginning at gestation week 13 and is maintained throughout fetal development [88]. Quantification of MDR1 mRNA in 12 fetal human livers has revealed significant increases from 15 to 27 weeks, with a subsequent decline at 42 weeks [95]. Interestingly, there is a transient mRNA “surge” in Mdr2 at birth in mice [22,90]. This increase in Mdr2 mRNA is attenuated in mice lacking the bile acid nuclear receptor, Fxr, suggesting that establishment of bile acid signaling, in part, stimulates Mdr2 up-regulation in neonatal mice [22]. Expression of the human ortholog MDR3 mRNA and protein in fetal livers is relatively low and most immunostaining is observed in the intracellular compartment, with only occasional canalicular staining at the plasma membrane [82]. At this time, it is unclear whether MDR3 mRNA is enhanced at birth similar to Mdr2.

The excretion of organic anions from the liver is primarily performed by the multidrug resistance-associated proteins (Mrps, Abcc gene family). Mrps are located on both the sinusoidal (Mrp/Abcc 1, 3, 4, 6) and canalicular (Mrp2/Abcc2) plasma membrane (reviewed in [96]). Mrp2 mRNA and protein as well as Mrp6 mRNA is detected in the fetal rat liver at gestation days 16–20 at approximately 10% of adult levels and increase to 27% to 40% by birth [81,91,97]. The timing of induction for these two transporters in mouse liver is somewhat different. The expression of Mrp2 mRNA in the fetal mouse liver is approximately 25% of that seen in the adult and reaches 100% at birth [92]. Similarly, human MRP2 mRNA is roughly 50% in fetal livers compared to adult livers [82]. Immunostaining of MRP2 protein in fetal hepatocytes has been demonstrated as primarily on the canalicular plasma membrane, consistent with adult localization [82].

In mice, Mrp6 mRNA is not detected until postnatal day 10. At this age, the expression of Mrp6 actually exceeds adult levels by 3-fold [92]. The amount of Mrp4 mRNA in the fetal mouse liver is similar to the adult, although there is a transient increase at birth [92,94]. By contrast, Mrp3 mRNA is low in the fetal liver and complete establishment is not observed until 20 to 30 days after birth [80,92,94]. Because of the divergent patterns in the expression of various hepatic Mrps, it is likely that there are differing regulatory mechanisms for each isoform.

The breast cancer resistance protein (Bcrp/Abcg2) is an additional efflux transporter that is responsible for the canalicular excretion of drugs and endogenous chemicals. Interestingly, Bcrp mRNA is highest in mouse and rat fetal livers and decreases during ontogeny [7,22]. This is an important observation and may reflect the fact that the fetal liver is primarily a hematopoetic tissue since Bcrp is an important marker of hematopoiesis [98].

### 4.3. Bile Acid Transporters

#### 4.3.1. Rodent and Human Bile Acid Transporter Regulation

Bile acid production and handling are initiated in mouse and rat liver at birth and increase steadily during the postnatal period [23,80]. Between postnatal day 1 and 45, taurocholate is the predominant bile acid in serum and liver [22]. The vectoral secretion of bile acids into bile is coordinated primarily by two transporters, the sinusoidal uptake transporter, sodium taurocholate co-transporting polypeptide (Ntcp/Slc10a1) and the canalicular efflux transporter, bile salt export pump (Bsep/Abcb11) [96] (Figure 1). Ntcp mRNA is absent through most of fetal development in the rat and is first detected at the end of gestation (gestation days 18–21) [99] when sodium-dependent uptake of the bile acid taurocholate can be quantified in vesicles prepared from rat liver sinusoidal plasma membranes [100]. Likewise, taurocholate uptake and efflux in a temperature-dependent manner can be observed in human hepatocytes cultured from fetal livers [83]. Gene expression of human BSEP and NTCP has been observed in fetal hepatocytes although at approximately 50% and less than 10% of the adult, respectively [83]. Following birth, Ntcp and Bsep mRNA and protein dramatically increase on postnatal days 0 to 1 in mice [80] and rats [81,84,97]. Both proteins can be seen just prior to birth, however, transporter immunostaining that is similar to adults is not observed until postnatal days 5 (Ntcp) and 12 (Bsep) [81]. It should be noted that other studies have not been able to detect Bsep protein in the fetal rat liver [91,97]. Similar to Ntcp and Bsep, the organic solute transporter beta (Ostβ), which transports bile acids in bile ducts, also exhibits a postnatal day 1 surge in expression in mice [22].

### 4.4. Absolute Transporter Expression in Fetal and Neonatal Livers

A recent study has quantified the absolute transcript levels of transporters in mouse liver using RNA-Sequencing and revealed that there is a similar number of uptake and efflux transporter mRNAs in the fetal liver (late gestation) [80]. At birth and over the following 3 weeks, mRNA levels of uptake transporters steadily increase, whereas only modest increases are observed in efflux transporter transcripts over the same time period [80]. Interestingly, the two most predominant transporter mRNAs in fetal mouse livers are Ent1 (29% of total transporter mRNAs) and Bcrp (25%) [80]. The absolute expression of other transporters in the fetal liver are modest and include Ntcp (6%), Mdr2 (6%), Oatp2a1 (4%), Mrp3 (3%), Mrp2 (3%), Mrp6 (2%), Bsep (2%) [80]. By parturition, there is a shift in the absolute expression of transporter mRNAs with Ntcp mRNA comprising 52% of total transporter mRNAs [80]. This is followed by Mdr2 (7%), Ent1 (6%), Mrp2 (5%), Bcrp (4%) and

Bsep (4%) [80]. Other transporters that appear to be enriched during the perinatal period include the multidrug and toxin extrusion 1 (Mate1) transporter, cholesterol transporter Abca1, the carnitine transporter Octn1, Oat3, Ent3 as well as Mrp1, 4, and 5.

### 4.5. Potential Ontogenic Mechanisms for Bile Acid Transporter Regulation

The marked increase in the expression of bile acid transporters at birth occurs in parallel with mRNA induction of transcription factors including Fxr (transcript variant 3) and its target gene, the short heterodimer partner, as well as Pxr [22]. Interestingly, mice lacking Fxr exhibit attenuated up-regulation of Ntcp, Bsep, and Ostβ mRNAs at birth suggesting a critical role for this transcription factor in the initiation of bile acid transporter expression [22]. Notably, no differences in bile acid transporter expression on postnatal day 1 were observed in Pxr-null mice [22], further supporting a more critical role for Fxr in the establishment of bile acid transport at birth.

## 5. Conclusions and Future Directions

**Figure 2.**
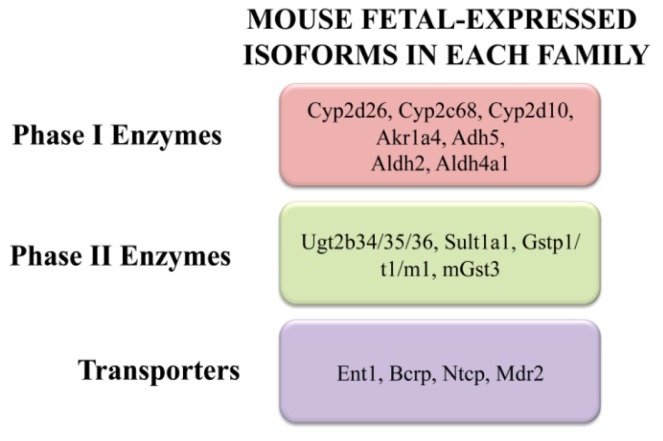
Enzymes and transporters expressed in the fetal mouse liver. RNA-sequencing studies have quantified the absolute mRNA expression of drug metabolizing and transport genes in the mouse fetal liver two days prior to birth [3,14,44,80].

The initiation and establishment of phase I, II, and transport pathways in the liver occurs in stages, often comprised of specific enzyme and transporter isoforms. Some enzymes and transporters exhibit modest expression in the fetal livers while others rapidly increase or “surge” at birth. Figure 2 illustrates the highest expressing enzymes and transporters based on RNA-Sequencing. Yet, others are not present until the postnatal, adolescent, or adult periods. As a result, there is immaturity of a number of key metabolic and disposition pathways during early development. Typically, the maternal excretory organs and the placenta will aid in protecting vulnerable fetuses until they begin to express a full complement of hepatic enzymes and transporters.

Current data suggest that the ontogeny of metabolizing enzymes and transporters is regulated by a combination of epigenetic (DNA methylation and histone modifications), transcriptional (nuclear receptors, transcription factors), and post-transcriptional mechanisms. Regulation can also be family-, subfamily-, or isoform-specific. Still, additional research is needed to more definitively understand these control mechanisms and to begin to probe novel regulator factors such as Hnfs, which are liver-specific transcription factors that develop early in gestation [41,101]. This will likely require greater utilization of translational approaches, including human fetal tissue banks as well human embryonic stem cells that can be differentiated into hepatocytes.

## Figures and Tables

**Table 1.** Phase I metabolism (non-cytochrome P450) in rodent and human fetal livers. 1

Enzyme	Mouse	Rat	Human	References
**Alcohol Dehydrogenases**				

Adh1	↓(M)			[3]
Adh4	↓(M)			[3]
Adh5	↓(M)			[3]
Adh6-ps1	↓(M)			[3]
Adh7	↓(M)			[3]

**Aldo-Keto Reductases**				

Akr1	↓(M)			[3]
Akr7	↓(M)			[3]

**Aldehyde Dehydrogenases**				

Aldh1	↓(M)			[2,3]
Aldh2	↓(M)			[2,3]
Aldh3a2	↓(M)			[2,3]
Aldh4a1	↓(M)			[2,3]
Aldh6a1	↓(M)			[2,3]
Aldh7a1	↓(M)			[2,3]

**Carboxylesterases**				

Ces		↓(A)		[7]
Ces1/CES1	↓(M)		↓(M,P,A)	[3,8]
Ces2/CES2	↓(M)		↓(M)	[3,8]
Ces3	↓(M)			[3]

**Flavin-Containing Monooxygenases**				

Fmo1/FMO1	↓(M)↔(P)		↑(M,P)	[3–6]
Fmo2	↓(M)			[3]
Fmo3/FMO3	↓(M,P)		↓(M)	[3–5]
Fmo4/FMO4	↔(M)		↔(M)	[3,5]
Fmo5	↓(M,P)			[3,4]

1 Expression or activity compared to adult liver levels. mRNA expression (M), protein expression (P), and activity (A) are noted; ↑ denotes up-regulation; ↓ denotes down-regulation; and ↔ denotes no change. Human orthologs are indicated only for isoforms with human data.

**Table 2.** Phase I metabolism (cytochrome P450) in rodent and human fetal livers. 1

Enzyme	Mouse	Rat	Human	References
**Cytochrome P450s**				

Cyp1a1/CYP1A1		↑(M)↓(A)	↓(M,P,A)	[7,12,13]
Cyp1a2/CYP1A2	↓(M)	↓(M,A)	↓(M,P,A)	[7,11–15]
Cyp1b1/CYP1B1	↓(M)		↑(M)	[11,13]
Cyp2a4	↓(M)			[11,14,15]
Cyp2a5	↓(M)			[14,15]
Cyp2a12	↓(M)			[14,15]
Cyp2a22	↓(M)			[14]
Cyp2b1		↓(M,A)		[7,16,17]
Cyp2b2		↓(M,A)		[7,16]
Cyp2b9	↓(M)			[14,15]
Cyp2b10	↓(M)			[11,14,15]
Cyp2b13	↓(M)			[14,15]
Cyp2b23	↓(M)			[14]
Cyp2c6		↓(M)		[16]
Cyp2c7		↓(M)		[17]
CYP2C9			↓(P,A)	[18]
CYP2C19			↓(P,A)	[18]
Cyp2c29	↓(M)			[14,15]
Cyp2c37	↓(M)			[14]
Cyp2c38	↓↔(M)			[14,15]
Cyp2c39	↓↔(M)			[14,15]
Cyp2c40	↓(M)			[14]
Cyp2c44	↓(M)			[14,15]
Cyp2c50	↓(M)			[14,15]
Cyp2c54	↓(M)			[14,15]
Cyp2c55	↓(M)			[14]
Cyp2c66	↓(M)			[11]
Cyp2c67	↓(M)			[14]
Cyp2c68	↓(M)			[14,15]
Cyp2c69	↓(M)			[14]
Cyp2c70	↓(M)			[14,15]
Cyp2d9	↓(M)			[14,15]
Cyp2d10	↓(M)			[14,15]
Cyp2d11	↓(M)			[14]
Cyp2d12	↓(M)			[14]
Cyp2d13	↓↔(M)			[14,15]
Cyp2d22	↓(M)			[11,14,15]
Cyp2d26	↓(M)			[14,15]
Cyp2d34	↓(M)			[14]
Cyp2d37-ps	↓(M)			[14]
Cyp2d40	↓(M)			[14]
Cyp2e1/CYP2E1	↓(M)	↓(M,A)	↓(M)	[7,11,13–15]
Cyp2f2	↓(M)			[11,14,15]
Cyp2g1	↓↔(M)			[14,15]
Cyp2j5	↓(M)			[14,15]
Cyp2j6	↓(M)			[11,14]
Cyp2r1/CYP2R1	↓(M)		↓(M)	[13,14]
Cyp2s1/CYP2S1			↓(M)	[13]
Cyp2u1/CYP2U1	↓(M)		↓(M)	[13,14]
Cyp2w1/CYP2W1			↑(M)	[13]
Cyp3a1		↓(M,A)		[7,16,17]
Cyp3a2		↓(♂M,A)↑(♀M)		[7]
Cyp3a11/CYP3A4	↓(M)	↓(M)	↓(M,P,A)	[11,14,15,19–24]
CYP3A5			↔(M,P,A)	[19,21]
Cyp3a13	↓(M)			[11,14,15]
Cyp3a16/CYP3A7	↑↔(M)		↑(M,P,A)	[11,14,15,19–21]
Cyp3a25	↓(M)			[11,14,15]
Cyp3a41a/b	↑(♂M) ↓(♀M)			[11,14,15]
Cyp3a44	↓(M)			[14,15]
Cyp3a59	↓(M)			[14]
Cyp4a1		↔(M)↓(♂A)↑(♀A)		[7]
Cyp4a10	↓(M)			[11,14,15]
Cyp4a12a/b	↓(M)			[14]
Cyp4a14	↓↔(M)			[14,15]
Cyp4a31	↓(M)			[14,15]
Cyp4a32	↓(M)			[14]
Cyp4b1/CYP4B1	↓(M)		↓(M)	[13,14]
Cyp4f13	↓↔(M)			[14,15]
Cyp4f14	↓(M)			[14,15]
Cyp4f15	↓(M)			[14,15]
Cyp4f16	↓(M)			[14]
Cyp4f18	↓↔(M)			[11,14]
Cyp4f39	↓(M)			[14]
Cyp4v3	↓(M)			[14,15]
Cyp4x1/CYP4X1	↓(M)		↓(M)	[13]
Cyp7a1	↓↔(M)	↓(M)		[14,15,22,23]
Cyp7b1	↓↔(M)			[14,15,22]
Cyp8b1	↓(M)	↓(M)		[14,15,22,23]
Cyp17a1	↓(M)			[14]
Cyp20a1	↓(M)			[14]
Cyp26a1	↓(M)			[14,15]
Cyp26b1	↓(M)			[14]
Cyp27		↓(M)		[23]
Cyp27a1	↓(M)			[14,15,22]
Cyp39a1	↓(M)			[14]
Cyp51	↓↔(M)			[14,15]

1 Expression or activity compared to adult liver levels. mRNA expression (M), protein expression (P), and activity (A) are noted; ↑ denotes up-regulation; ↓ denotes down-regulation; and ↔ denotes no change. Specific data for females (♀) or males (♂) are noted. Human orthologs are indicated only for isoforms with human data. No data is available for Cyp3a57, Cyp4f17, and Cyp4f40.

**Table 3.** Phase II metabolism in rodent and human fetal livers. 1

Enzyme	Mouse	Rat	Human	References
**Sulfotransferases**				

Sult1a1/SULT1A1	↓↔(M)		↔↓(P)↓(A)	[15,44–49]
SULT1A3			↑(P,A)	[46,47,49,50]
Sult1b1	↓(♂M)			[44]
Sult1c1/SULT1C1	↑(M)			[45]
Sult1c2/SULT1C2	↓(M)		↑(P)	[44,45,47]
Sult1d1	↓↔(M)			[15,44,45]
Sult1e1/SULT1E1	↔(♂M)		↑(M,P)↔(A)	[44,47,48,51]
Sult2a1/SULT2A1	↓(M)		↓(P,A)	[44,45,47,48,52,53]
Sult3a1	↓(♀M)↔(♂M)			[15,45]
Papss2	↓(♀M)↔(♂M)			[45]

**Glutathione*S*-transferases**				

Gsta1/2/GSTA1/2	↓(M)	↓(P)	↓(P)	[15,44,54–57]
Gsta3	↓(M)			[15,44,54]
Gsta4	↓(M)			[44,54]
Gsta8		↓(P)		[55]
Gsta10		↑(P)		[55]
Gstk1	↓(M)			[15,54]
Gstm1/GSTM1	↓↔(M)	↓(P)	↑(P)	[15,44,54–56]
Gstm2	↓(M)			[15,44,54]
Gstm3	↓↔(M)	↓(P)		[15,44,54,55]
Gstm4	↓(M)	↓(P)		[15,44,54,55]
Gstm5	↑(M)			[15,44,54]
Gstm6	↓↔(M)			[15,44,54]
Gstm7	↓(♂M)			[44]
Gsto1	↓(M)			[44,54]
Gstp1/2/GSTP1	↓(M)		↑(P)	[15,44,54,56,57]
Gstp7		↑(P)		[55]
Gstt1	↓↔(M)			[15,44,54]
Gstt2	↔(M)			[15,44,54]
Gstt3	↓↔(M)			[15,44,54]
Gstz1/GSTZ1	↓(M)		↓(P,A)	[15,44,54,56]
Mgst1	↓(M)			[15,44]
Mgst2	↑↔(M)			[44,54]
Mgst3	↓↑(M)			[15,44,54]

**UDP Glucuronosyltransferases**				

Ugt1a1/UGT1A1	↓(♂M)	↓(M,P,A)	↓(A)	[44,58,59]
UGT1A3			↓(A)	[59]
Ugt1a5	↓(♂M)	↓(M,P)		[44,58]
Ugt1a6/UGT1A6	↓(♂M)	↑(M,P)	↓(A)	[44,58,60]
Ugt1a9	↓(♂M)			[44]
Ugt2a3	↓(M)			[15,44]
Ugt2b1	↓(M)			[15,44]
Ugt2b5	↓(M)			[15,44]
UGT2B7			↓(M,A)	[61,62]
UGT2B15			↓(M)	[61]
UGT2B17			↓(M,A)	[61,63]
Ugt2b34	↓↔(M)			[15,44]
Ugt2b35	↓(M)			[15,44]
Ugt2b36	↓(M)			[15,44]
Ugt2b37	↓↔(M)			[15,44]
Ugt2b38	↓(♂M)			[44]
Ugt3a1	↓↔(M)			[15,44]
Ugt3a2	↓(M)			[15,44]

***N*-Acetyltransferases**				

Nat1/NAT1	↓(M,A)		↔(A)	[44,64,65]
Nat2	↓(M,A)			[44,64]
Nat8	↓(♂M)			[44]

1 Expression or activity compared to adult liver levels. mRNA expression (M), protein expression (P), and activity (A) are noted; ↑ denotes up-regulation; ↓ denotes down-regulation; and ↔ denotes no change. Specific data for females (♀) or males (♂) are noted. Human orthologs are indicated only for isoforms with human data.

**Table 4.** Transport in rodent and human fetal livers. 1

Transporter	Mouse	Rat	Human	References
**Uptake Transporters**				

Slc1a5	↑(M)			[15]
Slc2a1	↑(M)			[15]
Slc2a3	↑(M)			[15]
Slc3a2	↑(M)			[15]
Slc4a1	↑(M)			[15]
Slc6a9	↔(M)			[15]
Slc7a1	↑↔(M)			[15]
Slc7a5	↑(M)			[15]
Slc10a1/Ntcp/NTCP	↓(M,P)	↓(M,P)	↓(M)	[22,80–85]
Slc10a2/Asbt	↓(M)			[80]
Slc14a1	↑(M)			[15]
Slc16a1	↑(M)			[15]
Slc17a1/Npt1	↓(M)			[80]
Slc20a1	↑(M)			[15]
Slc22a1/Oct1	↓(M)	↓(M)		[7,22,80,86]
Slc22a2/Oct2		↑(♀M)↓(♂M)		[7]
Slc22a4/Octn1	↑(M)			[80]
Slc22a5/Octn2	↔(M)			[80]
Slc22a6/Oat1		↓(M)		[7]
Slc22a7/Oat2	↓(M)	↓(M)		[7,22,80,85]
Slc22a8/Oat3	↑(M)	↑(M)		[7,80]
Slc25a4	↑(M)			[15]
Slc25a37	↑(M)			[15]
Slc25a38	↔(M)			[15]
Slc29a1/Ent1	↔(M)			[22,80]
Slc29a3/Ent3	↔(M)			[80]
Slc38a1	↑(M)			[15]
Slc38a5	↑(M)			[15]
Slc39a5	↑(M)			[15]
Slc39a8	↔(M)			[15]
Slc43a1	↑(M)			[15]
Slc43a3	↑(M)			[15]
Slc47a1/Mate1	↓(M)			[22,80]
Slc51a/Ostα	↔(M)			[22]
Slc51b/Ostβ	↔(M)			[22,80]
Slco1a1/Oatp1a1	↓(M)	↓(M,♂P)		[22,80,81,85,87]
Slco1a4/Oatp1a4	↓↔(M)	↑↓(M)↓(♂P)		[7,22,80,81,87]
Slco1a6/Oatp1a6	↔(M)			[87]
SLCO1B1/OATP1B1			↓(M)	[83]
Slco1b2/Oatp1b2	↓(M)	↓(M,♂P)		[22,80,81,85,87]
SLCO1B3/OATP1B3			↓(M)	[83]
Slco4a1/Oatp4a1		↓(M)		[85]
Slco2a1/Oatp2a1	↓↔(M)			[80,87]
Slco2b1/Oatp2b1/2B1	↓(M)	↓(M)	↓(M)	[22,80,83,85,87]

**Efflux Transporters**				

Abca1	↑↔(M)			[22,80]
ABCB1/MDR1			↓(M,P)	[83,88]
Abcb1a/Mdr1a		↓↔(M)		[7,89]
Abcb1b/Mdr1b	↔(M)	↓(M)		[7,89,90]
Abcb4/Mdr2/MDR3	↓↔(M)		↓(M,P)	[22,80,82,83,90]
Abcb10	↑(M)			[15]
Abcb11/Bsep/BSEP	↓↔(M)↓(P)	↑↓(M,P)	↓(M,P)	[7,22,80–83,85,91]
Abcc1/Mrp1	↑(M)	↑(M)		[7,80,85,89]
Abcc2/Mrp2/MRP2	↓(M)	↓↔(M,P)	↓(M,P)	[7,22,80–83,85,89,91,92]
Abcc3/Mrp3/MRP3	↓(M)	↑↔(M)	↓(M)	[7,22,80,83,85,92]
Abcc4/Mrp4/MRP4	↑↔(M)		↔(M)	[22,80,83,92]
Abcc5/Mrp5	↑(M)			[80]
Abcc6/Mrp6	↓(M)	↑↓(M)↓(♂P)		[7,22,80,81,85,92]
Abcg2/Bcrp/BCRP	↑↔(M)	↑(M)	↓↔(M)	[7,15,22,80,83]
Abcg5	↓↔(M)			[22,80]
Abcg8	↔(M)			[22,80]
Atp7b	↑(M)			[80]
Atp8b1	↓(M)			[22]

1 Expression or activity compared to adult liver levels. mRNA expression (M), protein expression (P), and activity (A) are noted; ↑ denotes up-regulation; ↓ denotes down-regulation; and ↔ denotes no change. Specific data for females (♀) or males (♂) are noted. Human orthologs are indicated only for isoforms with human data.

## Abbreviations

Abc: ATP-binding cassette
Adh: alcohol dehydrogenase
Akr: aldo-keto reductase
Aldh: aldehyde dehydrogenase
Bcrp: breast cancer resistance protein
Bsep: bile salt export pump
Car: constitutive androstane receptor
Ces: carboxylesterase
Cyp: cytochrome P450
Ent: equilibrative nucleoside transporter
Fgf: fibroblast growth factor
Fmo: flavin-containing monooxygenase
Fxr: farnesoid X receptor
Gst: glutathione *S*-transferase
Hnf: hepatic nuclear factor
Lxr: liver X receptor
Mate: multidrug and toxin extrusion
Mdr: multidrug resistance
Mrp: multidrug resistance-associated protein
Nat: *N*-acetyl transferase
Ntcp: sodium taurocholate co-transporting polypeptide
Oat: organic anion transporter
Oatp: organic anion transporting polypeptide
Oct: organic cation transporter
Ost: organic solute transporter
Ppar: peroxisome proliferator-activated receptor
Pxr: pregnane X receptor
Slc: solute carrier
Sult: sulfotransferase
Ugt: UDP-glucuronosyl transferase
